# Curcumin-mediated resilience to mixed *Eimeria* challenge in broilers fed soybean or canola oil: growth performance, hematology, coccidial lesions, oocyst shedding, digestibility, and intestinal barrier biology

**DOI:** 10.1016/j.psj.2026.106981

**Published:** 2026-04-19

**Authors:** Hussein Maytham Abdulhusein, Kamran Taherpour, Hossein Ali Ghasemi, Hassan Shirzadi, Fatemeh Tavakolinasab

**Affiliations:** a Department of Animal Science, Faculty of Agriculture, Ilam University, Ilam, Iran; b Department of Animal Science, Faculty of Agriculture and Environment, Arak University, 38156-8-8349 Arak, Iran

**Keywords:** Coccidiosis-challenged broilers, Curcumin, Fat source, Gut health, Performance

## Abstract

A 42-day study evaluated whether dietary fat source (soybean oil vs. canola oil) and curcumin supplementation enhance broiler resilience to coccidiosis. In total, 660 Ross 308 male broilers were assigned to a completely randomized design with five treatments (6 replicates per treatment; 22 birds per replicate): (1) unchallenged control with soybean oil; (2) soybean oil + coccidiosis challenge; (3) soybean oil + 0.02% curcumin + challenge; (4) canola oil + coccidiosis challenge; and (5) canola oil + 0.02% curcumin + challenge. On day 14, challenged birds received a 1 mL oral gavage of sporulated oocysts (50,000 *Eimeria acervulina*, 10,000 *E. maxima*, and 5,000 *E. tenella* per bird), whereas unchallenged birds received sterile saline. The challenge impaired growth and feed efficiency and increased mortality, accompanied by higher intestinal lesion scores and oocyst shedding, reduced nutrient utilization, disrupted villus–crypt architecture and goblet cell counts, and downregulated jejunal barrier-associated transcripts (*P* < 0.05). Curcumin (0.02%) mitigated these effects by improving body weight at days 24 and 42, increasing average daily gain during days 11–24 and across days 1–42, improving feed conversion ratio during days 11–24 and overall, reducing mortality, and increasing the European performance index (*P* < 0.05). Curcumin also reduced duodenal and cecal lesion severity, decreased fecal oocyst shedding across post-challenge intervals, improved crude protein and ash digestibility, and restored intestinal morphology (greater villus height and surface area, a higher villus height-to-crypt depth ratio, and increased goblet cell counts) (*P* < 0.05). Hematological measures further supported improved resilience, with lower heterophil counts and heterophil-to-lymphocyte ratio and higher hematocrit (*P* < 0.05). At the molecular level, curcumin upregulated jejunal CLDN1 and MUC2, whereas OCLN and ZO1 were unchanged (*P* < 0.05). In conclusion, these findings show that 0.02% curcumin strengthens intestinal integrity and reduces parasite burden, translating into measurable improvements in flock-level performance under coccidiosis challenge and supporting its practical use as a nutrition-based strategy to enhance disease resilience.

## Introduction

Coccidiosis remains one of the most consequential enteric diseases in modern broiler production because it couples direct intestinal injury with a substantial inflammatory and oxidative burden that may persist beyond the acute phase (Mishra and Jha, 2019; Razavi et al., 2024). During *Eimeria* infection, epithelial integrity and absorptive capacity are compromised and endogenous nutrient losses increase; concurrently, immune activation redirects energy and amino acids away from growth (Mathis et al., 2025; Chen et al., 2025). As a result, coccidiosis often manifests as a multifactorial enteric disorder characterized by reduced weight gain and feed efficiency, altered hematological indices, intestinal lesions, and disruption of intestinal barrier biology (Biabani et al., 2024a; Teng et al., 2020). Although anticoccidial drugs and vaccination remain essential, variable field responses and increasing pressure to reduce chemotherapeutic reliance have intensified interest in nutritional strategies and functional feed additives that strengthen gut resilience during enteric challenge (Ayalew et al., 2022; Rostami et al., 2021; Zhu et al., 2021).

Among candidate additives, curcumin (from *Curcuma longa*) has received sustained attention because it can modulate oxidative stress and inflammatory signaling, central features of coccidiosis pathogenesis, while supporting intestinal structure and function (Petrone-Garcia et al., 2021; Yadav et al., 2020). Recent syntheses of broiler studies commonly associate curcumin supplementation with improved growth performance and antioxidant status, alongside favorable changes in intestinal morphology and related health indicators (Aderemi and Olufemi, 2023; Fouad et al., 2025). Controlled experiments further indicate that curcumin can attenuate oxidative damage under pro-oxidant conditions, supporting its relevance when intestinal injury and reactive oxygen species production increase during enteric stress (Oke et al., 2024; Wu et al., 2023). Accordingly, phytogenic feed additives, including curcumin-based approaches, are increasingly integrated into non-antibiotic intestinal health programs aimed at preserving barrier function and productive efficiency under infectious pressure (Alharbi et al., 2025; Wang et al., 2024).

However, the nutritional context in which phytogenics are delivered can shape the magnitude and consistency of responses, and dietary lipid source is a particularly relevant component of that context. Added fats increase dietary energy density and influence digestion and metabolism, but differences in fatty acid profile can also alter membrane composition, bile acid dynamics, microbial ecology, and downstream immune tone (Jadhav et al., 2024; Mohammadi and Rudkowska, 2025). This distinction is particularly relevant when comparing soybean and canola oils: although both are unsaturated vegetable oils, they differ in their omega-6:omega-3 balance and monounsaturated fatty acid content, differences that may lead to divergent physiological responses and product quality outcomes (Elbaz et al., 2023). Evidence from early-life nutrition further indicates that oil source can influence lipid utilization and digestive development, reinforcing that fat source is not biologically interchangeable (Akbari Moghaddam Kakhki et al., 2025). The unsaturated nature of soybean and canola oils also raises a practical concern, susceptibility to lipid peroxidation and the formation of lipid oxidation products (Ansorena et al., 2023). Such products can depress performance and reduce energy value, and may exacerbate intestinal and systemic oxidative load, an especially undesirable scenario during coccidiosis, when oxidative stress is already elevated (Kerr et al., 2025; Razavi et al., 2024). In this context, antioxidants may be strategic rather than merely supportive, by limiting oxidation-driven inefficiencies and helping stabilize barrier function and immune homeostasis (Mahfuz et al., 2021). Curcumin is therefore of particular interest in diets containing high-UFA oils, because its antioxidant capacity may be most valuable when unsaturated lipid matrices increase peroxidation risk and when enteric challenge amplifies oxidative injury (Hernández-García et al., 2025; Wu et al., 2023).

We hypothesized that curcumin would mitigate coccidiosis-associated penalties in performance and gut health, and that responses could differ by fat source because dietary lipids shape the microbial and metabolic milieu and influence oxidative susceptibility, thereby affecting the expression of an antioxidant phytogenic under enteric challenge. Accordingly, this study compared soybean oil and canola oil, with or without 0.02% curcumin, in broiler chickens exposed to a coccidiosis challenge. Production-relevant outcomes were integrated with mechanistically informative endpoints, including growth performance indices, hematology and leukocyte differentials, intestinal lesion scores and oocyst shedding, nutrient digestibility, gut morphometry, and jejunal barrier-related gene expression.

## Materials and methods

### Animal ethics

All animal procedures were conducted in accordance with established guidelines for the care and use of experimental animals and followed the general experimental design and husbandry practices (Biabani et al., 2024a,2024b). The protocol was reviewed and approved by the Animal Ethics Committee of Ilam University (approval no. IR.ILAM.REC.1405.001). Birds were monitored at least twice daily, and any welfare concerns were addressed immediately in accordance with the approved protocol.

### Birds, treatments and management

A total of 660 one-day-old male Ross 308 broiler chicks were used in a 42-day experiment arranged as a completely randomized design with five treatments, each comprising six replicates of 22 birds. Birds were housed in litter-floor pens (approximately 1.5 × 1.8 m) with ad libitum access to feed and water. Standard environmental management was applied, including uniform lighting and adequate ventilation; house temperature was maintained at 34 ± 1°C during the first week and gradually reduced to 23 ± 1°C thereafter. The five experimental treatments were as follows: (1) an unchallenged negative control fed a soybean-oil diet and gavaged with sterile saline (**NC**); (2) a challenged control fed a soybean-oil diet and orally challenged with a mixed *Eimeria* spp. inoculum (**PC+SO**); (3) a challenged group fed a soybean-oil diet supplemented with 0.02% curcumin (200 mg/kg) and challenged with a mixed *Eimeria* spp. inoculum (**PC+SO+CUR**); (4) a challenged group fed a canola-oil diet and challenged with a mixed *Eimeria* spp. inoculum (**PC+CO**); and (5) a challenged group fed a canola-oil diet supplemented with 0.02% curcumin and challenged with a mixed *Eimeria* spp. inoculum (**PC+CO+CUR**).

Curcumin, a 95% natural turmeric extract, was procured from Karen Company (Yazd, Iran) for this study. Curcumin incorporated by premixing with a small quantity of the basal feed, followed by thorough blending into the complete diet to ensure homogeneous distribution. Soybean and canola oils used in diet formulation were analyzed for physicochemical quality (AOCS, 2011) and fatty acid composition (Table 1). Soybean oil contained a higher proportion of total polyunsaturated fatty acids (PUFA; 58.9%) and a higher n-6/n-3 PUFA ratio (15.83) than canola oil (PUFA, 36.0%; n-6/n-3, 4.54), whereas canola oil had a substantially higher monounsaturated fatty acid (MUFA) content (57.03%) than soybean oil (24.94%). Both oils exhibited low free fatty acid contents (0.205% and 0.181%) and low peroxide values (3.12 and 4.20 mEq/kg for soybean and canola oils, respectively). Diets were provided in three phases (starter, 0–10 d; grower, 11–28 d; finisher, 29–42 d) and were formulated as corn–soybean meal–based rations to meet broiler nutrient requirements (Table 2). All basal diets contained added oil at 40.0 g/kg (as-fed) across phases. Consistent with the oil profiles (Table 1), analyzed dietary fat content and fatty acid patterns in the starter, grower, and finisher diets showed higher C18:2n-6 concentrations and higher n-6/n-3 ratios in soybean-oil diets than in canola-oil diets (Table S1).

**Table 1:** Physicochemical properties and fatty acid profile of soybean oil and canola oil used in broiler diets.

Item^1^	Soybean oil	Canola oil
Moisture (%)	0.055	0.042
Impurities (%)	0.037	0.041
Unsaponifiable material (%)	1.03	1.22
Free fatty acid (%)	0.205	0.181
Peroxide value (mEq/kg)	3.12	4.20
Gross energy, kcal/kg	9385	9410
Fatty acid profile (Relative %)		
C14:0	0.102	0.052
C16:0	11.4	4.7
C18:0	4.1	1.4
C20:0	0.255	0.43
Total SFA	15.86	6.58
C16:1	0.13	0.51
C18:1	24.5	55.1
C20:1	0.31	1.42
Total MUFA	24.94	57.03
C18:2n-6 (LA)	55.4	29.5
C18:3n-3 (ALA)	3.5	6.5
Total PUFA	58.9	36
n-6/n-3 PUFA	15.83	4.54

Abbreviations: SFA, saturated fatty acid (Sum of C14:0, C16:0, C17:0, C18:0, and C20:0); MUFA, Monounsaturated fatty acid (Sum of C16:1, C18:1, and C20:1); LA, linoleic acid; ALA, α-linolenic acid; PUFA, polyunsaturated fatty acid (Sum of C18:2n-6 and C18:3n-3).

**Table 2:** Ingredient and chemical composition of basal diet (g/kg on an as fed basis).

Item	0−10 d	11–28 d	29–42 d
Ingredient			
Corn	509.4	565.8	632.8
Soybean meal (44% CP)	396.6	320.9	242.4
Corn gluten meal (60% CP)	7.6	29.7	43.4
SO or CO^1^	40.0	40.0	40.0
Dicalcium phosphate	19.1	17.3	15.7
Limestone	11.3	10.7	10.1
Common salt	2.5	2.2	1.9
Sodium bicarbonate	0.5	1.0	1.5
DL-Methionine	2.8	2.5	2.1
L-Lysin, HCL	3.2	3.2	3.6
L-Threonine	2.0	1.7	1.5
Vitamin premix^2^	2.5	2.5	2.5
Mineral premix^3^	2.5	2.5	2.5
Calculated composition			
Metabolizable energy, kcal/kg	3000	3100	3200
Crude protein, g/kg	230	215.0	195.0
Calcium, g/kg	9.6	8.8	8.0
Available phosphorus, g/kg	4.8	4.4	4.0
Digestible lysine, g/kg	12.8	11.5	10.3
Digestible total sulfur amino acids, g/kg	9.5	8.7	8.0
Digestible threonine, g/kg	8.6	7.8	6.9
DEB^4^, mEq/100 g	250	235	225

1 SO, soybean oil; CO, canola oil.

2 Supplies per kg of the diet: vitamin A (retinyl acetate), 11000 IU; vitamin D_3_ (cholecalciferol), 1800 IU; vitamin E (DL-α-tocopheryl acetate), 11 mg; vitamin K_3_ (menadione dimethylpyrimidinol), 2 mg; thiamin (thiamine mononitrate), 1.6 mg; riboflavin, 6 mg; niacin, 30 mg; d-calcium pantothenate, 15 mg; pyridoxine, 2 mg; biotin, 0.25 mg; folic acid, 0.8 mg; vitamin B_12_, 0.020 mg; choline (choline chloride), 500 mg.

3 Supplies per kg of the diet: Mn (manganese oxide), 80 mg; Zn (zinc sulfate), 80 mg; Fe (ferrous sulfate), 35 mg; Cu (cupric sulfate), 10 mg; I (potassium iodide), 1 mg; Se (sodium selenite), 0.30 mg.

4 DEB (dietary electrolyte balance) = (Na^+^, mEq/kg + *K*^+^, mEq/kg) – CL^−^, mEq/kg.

### Coccidia species challenge

Coccidiosis was induced on day 14 in all challenged treatments using an oral-gavage model. Each bird in the challenged groups received 1 mL of a suspension containing sporulated oocysts of mixed *Eimeria* spp., delivering approximately 50,000 *Eimeria acervulina*, 10,000 *E. maxima*, and 5,000 *E. tenella* oocysts per bird (Biabani et al., 2024a,2024b). Oocysts were obtained from the Parasitology Laboratory, University of Tehran (Tehran, Iran). The field isolates were originally collected from feces, litter, and intestinal samples from commercial broiler farms and maintained in potassium dichromate solution to allow sporulation before preparation of the challenge inoculum. To standardize handling stress, birds in the unchallenged NC group received 1 mL of sterile saline by oral gavage on day 14.

### Growth performance measurements

Body weight (**BW**) was recorded on a pen basis at 1, 10, 24, and 42 d of age by weighing all birds within each replicate pen. Feed intake was determined for each pen over the corresponding intervals by recording feed offered and feed refusals at each weighing point. Body weight gain (**BWG**) was calculated for each period (1–10, 11–24, and 25–42 d) and cumulatively (1–42 d) as the difference between initial and final pen BW for the respective interval. Average daily feed intake (**ADFI**) was computed as pen feed consumption divided by the number of days in each interval and expressed on a per-bird, per-day basis using the mean number of live birds in the pen during that period. Feed conversion ratio (**FCR**) was calculated for each interval and overall as ADFI/ADG. Mortality was recorded daily, and performance variables were corrected for mortality. The European Performance Index (**EPI**) was calculated for the overall period (1–42 d) according to Ahmadi-Sefat et al. (2022) as follows: EPI = [Livability (%) × Live weight (kg) × 100] ÷ [Age (d) × FCR].

### Hematological indices

On day 24, two birds per replicate pen (selected to be close to the pen mean BW) were chosen for blood collection. Approximately 2 mL of blood was obtained from the brachial (wing) vein into EDTA-coated tubes. Red blood cell (**RBC**) and white blood cell (**WBC**) counts were determined using a hemocytometer with Natt–Herrick solution as the diluent and stain (Natt and Herrick, 1952). Hematocrit (%) was measured using the microhematocrit method, and hemoglobin concentration was determined by the cyanmethemoglobin technique (Keçeci et al., 1998). For leukocyte differentials, blood smears were examined under a light microscope and 100 leukocytes per sample were classified to determine the relative proportions of heterophils and lymphocytes, following Lucas and Jamroz (1961). The heterophil-to-lymphocyte (**H/L**) ratio was calculated for each bird as an indicator of physiological stress.

### Intestinal Lesion scores and oocyst shedding

At 6 days post-challenge (day 20), two broilers per replicate pen with body weight close to the pen mean were selected and euthanized for lesion assessment, following established mixed-*Eimeria* challenge procedures (Nooreh et al., 2021). The small intestine and ceca were opened longitudinally, and gross lesions were evaluated in segments corresponding to the primary predilection sites of the challenge species (duodenum, jejunum, and cecum). Lesions were scored on a standardized 0–4 ordinal scale (0 = no visible lesions; 4 = most severe lesions) by the same trained evaluator, blinded to treatment allocation (Johnson and Reid, 1970).

Fresh droppings were collected from each pen during 6–8, 8–10, and 10–12 days post-infection (**dpi**) (days 20–26). Within each interval, multiple fresh excreta samples per pen were pooled to obtain a representative pen-level sample and to reduce the influence of short-term variation in oocyst excretion (Bortoluzzi et al., 2025). Within each interval, pooled samples were homogenized, and a representative subsample was stored at 4°C until analysis. Oocyst shedding was quantified using the McMaster method. Briefly, a 5-g excreta aliquot was mixed with 45 mL of saturated salt solution in a 50-mL tube, vortexed to homogenize, and loaded into a McMaster chamber. After standing at room temperature for 5 min, oocysts were counted microscopically and expressed as oocysts per gram of excreta (OPG), as previously described (Ghasemi et al., 2010; Nooreh et al., 2021).

### Digestibility assay

Apparent ileal digestibility (**AID**) of nutrients was determined using an indigestible-marker approach as previously described (Nasiri et al., 2025). Chromium oxide (Cr₂O₃) was incorporated into all experimental diets from day 19 to day 24 at 0.3% (3 g/kg) to allow marker equilibration and stable recovery. On day 24, three birds per replicate pen were randomly selected and humanely slaughtered. Ileal digesta were collected from the intestinal segment extending from the distal jejunum to the ileocecal valve, pooled within pen, and prepared for laboratory analysis. Representative samples of feed and ileal digesta were dried (55–60°C), ground, and analyzed for dry matter, crude protein, crude fat, and ash using standard methods (AOAC International, 2011). Chromium concentrations in feed and digesta were determined by UV–Vis spectrophotometry (Shimadzu UV-1201, Japan) according to the chromic oxide procedure described by Mueller (1956). AID of each nutrient was calculated using the marker-ratio equation:

AID (%) = [(Marker in feed, %) / (Marker in digesta, %)] × [(Nutrient in digesta, %) / (Nutrient in feed, %)] × 100.

Marker-based nitrogen-corrected apparent metabolizable energy (**AMEn**) was also determined. Fresh excreta were collected from each pen twice daily from day 20 to day 24 using clean plastic trays placed beneath the pens, pooled by pen across collection days, and processed for analysis. Gross energy of diets and dried excreta was measured by bomb calorimetry (Parr Instrument Company, Moline, IL). Nitrogen output for correction was derived from crude protein analysis (AOAC International, 2011), and AMEn was calculated using the marker method with nitrogen correction, consistent with Nasiri et al. (2025).

### Intestinal morphology

On day 24, intestinal morphometry was assessed using a protocol consistent with recent mixed-*Eimeria* challenge studies (Ghasemi et al., 2025). Two birds per replicate pen with body weight close to the pen mean were euthanized, and approximately 1-cm segments were collected from the mid-duodenum, mid-jejunum, and mid-ileum. Samples were immediately fixed in buffered formaldehyde, processed using standard histological procedures, embedded in paraffin, and sectioned at approximately 5 μm. Sections were stained with Alcian blue, hematoxylin, and eosin, and examined by light microscopy (Olympus CX31, Shinjuku, Tokyo, Japan). Quantitative measurements were obtained using QWinPlus software (v3.1.0, Leica Cambridge Ltd., Cambridge, UK).

For each intestinal segment, villus height (**VH**), villus width (**VW**), and crypt depth (**CD**) were measured from well-oriented villus–crypt units, and the VH:CD ratio was calculated. Villus surface area (**VSA**) was estimated using a cylindrical approximation, VSA = 2π × (VW/2) × VH. For each sample, mean morphometric values were calculated from 10 intact villi. Goblet cells (**GC**) were quantified by selecting 15 straight, intact villi with their associated crypts per sample and counting goblet cells microscopically; results were expressed as GC per selected villus–crypt unit set, following the described counting strategy (Ghasemi et al., 2025).

### Gene expression of tight junction proteins and MUC2

On day 24, a mid-jejunal segment from two euthanized birds per replicate pen was excised, gently rinsed with sterile saline, snap-frozen in liquid nitrogen, and stored at −80°C until analysis. Total RNA was extracted from jejunal tissue using a commercial RNA extraction kit (Pars Tous Co., Iran) according to the manufacturer’s instructions. RNA concentration and purity were assessed using a NanoDrop 2000 spectrophotometer (Thermo Fisher Scientific, Waltham, MA, USA). Complementary DNA (cDNA) was synthesized from equal amounts of RNA using a cDNA synthesis kit (Pars Tous, Iran). Quantitative real-time PCR (qPCR) was performed in duplicate using an ABI 7300 Real-Time PCR System (Applied Biosystems, Foster City, CA, USA) with SYBR Green master mix (Pars Tous, Iran), following previously described procedures and reporting standards and in accordance with MIQE guidelines (Bustin et al., 2009, Mohammadi et al., 2025). Transcripts of junctional adhesion molecule 2 (**JAM2**), claudin-1 (**CLDN1**), occludin (**OCLN**), zonula occludens-1 (**ZO1**), and mucin-2 (**MUC2**) were quantified, with GAPDH used as the reference gene. Primer sequences, amplicon lengths, and GenBank accession numbers are provided in Table S2. Relative expression was calculated using the 2^−ΔΔCt^ method, in which target-gene Ct values were normalized to GAPDH and expressed relative to the NC group (Livak and Schmittgen, 2001).

### Statistical analysis

All data were analyzed using SAS software (v9.4; SAS Institute Inc., Cary, NC, USA). Data distributions were evaluated using the Shapiro–Wilk test to assess normality. For the primary treatment comparisons, data were analyzed as a completely randomized design with five treatments and six replicates per treatment, with pen as the experimental unit. When the treatment effect was significant, means were separated using Tukey’s multiple comparison procedure. To further partition dietary effects among challenged birds, a 2 × 2 factorial analysis was conducted after excluding the unchallenged NC group. This model tested the main effects of fat source (soybean oil vs. canola oil), curcumin (0 vs. 0.02%), and their interaction (fat source × curcumin), and the corresponding *P*-values were reported to aid interpretation of independent and interactive effects. Because lesion scores did not meet parametric assumptions, they were analyzed using the Kruskal–Wallis H test. Statistical significance was declared at *P* < 0.05, and 0.05 ≤ *P* < 0.10 was considered a statistical tendency.

## Results

### Growth performance

Growth performance results are summarized in Table 3. BW through 10 d was not affected by treatment (*P* > 0.05). At 24 and 42 d, BW was higher in the PC+CO+CUR group, which did not differ from the NC, compared with the PC+SO and PC+CO challenged groups (*P* < 0.05). Moreover, BW at 24 and 42 d was higher in the PC+SO+CUR group than in the NC (*P* < 0.05). ADG from 11 to 24 d was greater in the NC and PC+CO+CUR groups than in the PC+SO and PC+CO groups (*P* < 0.05). From 25 to 42 d, ADG was higher in the NC than in the PC+SO group (*P* < 0.05). Over the full period (1–42 d), ADG was higher in the NC and PC+CO+CUR groups than in the PC+SO and PC+CO groups, and PC+SO+CUR also increased ADG relative to PC+SO (*P* < 0.05). ADFI during 11–24 and 25–42 d was lower in the PC+SO and PC+SO+CUR groups than in the NC group, whereas the PC+CO and PC+CO+CUR groups were intermediate; these latter groups differed significantly only from PC+SO for ADFI during 11–24 d (*P* < 0.05). Across 1–42 d, all challenged treatments had lower ADFI than the NC, with the lowest intake observed in the PC+SO group (*P* < 0.05). FCR during 1–10 and 25–42 d was not affected by treatment (*P* > 0.05). However, during 11–24 d, FCR was lower in the NC than in the PC+CO group (*P* < 0.05). Overall (1–42 d), FCR was lower in the NC, PC+SO+CUR, and PC+CO+CUR groups than in the PC+CO group (*P* < 0.05). Among challenged groups, PC+CO+CUR exhibited the lowest mortality and the highest EPI relative to PC+SO and PC+CO (*P* < 0.05), and these values were comparable to the NC. In contrast, the NC group had lower mortality and a higher EPI than PC+SO+CUR (*P* < 0.05).

**Table 3:** Effects of dietary fat source and curcumin supplementation on growth performance observed in broiler chickens infected with a mixture of *Eimeria* species at 14 d of age.

	Treatments^1^							*P*-values (2 × 2 factorial design)		
Item	NC	PC+SO	PC+SO+CUR	PC+CO	PC+CO+CUR	SEM	*P*-values^2^	Fat source	CUR	Fat × CUR
BW, g										
10 d	234.9	234.4	239.9	235.6	240.4	4.09	0.742	0.842	0.207	0.934
24 d	1142^a^	990^d^	1062^bc^	1006^cd^	1082^ab^	16.6	<0.001	0.322	0.001	0.882
42 d	2923^a^	2627^d^	2751^bc^	2665^cd^	2818^ab^	29.8	<0.001	0.110	<0.001	0.661
ADG, g/bird/d										
1-10 d	19.09	19.05	19.54	19.15	19.63	0.405	0.753	0.819	0.228	0.983
11-24 d	64.76^a^	53.98^c^	58.69^bc^	55.01^c^	60.12^ab^	1.206	<0.001	0.350	0.001	0.874
25-42 d	98.98^a^	90.93^b^	93.85^ab^	92.20^ab^	96.42^ab^	1.890	0.039	0.358	0.092	0.749
1-42 d	68.55^a^	61.50^d^	64.44^bc^	62.41^cd^	66.04^ab^	0.712	<0.001	0.111	<0.001	0.662
ADFI, g/bird/day										
1-10 d	22.54	22.40	22.87	22.94	22.63	0.447	0.897	0.758	0.842	0.399
11-24 d	93.29^a^	85.14^c^	89.74^b^	90.18^ab^	90.35^ab^	0.833	<0.001	0.002	0.007	0.012
25-42 d	192.4^a^	181.6^b^	184.1^b^	187.5^ab^	186.9^ab^	1.522	0.001	0.012	0.562	0.360
1-42 d	118.9^a^	111.6^c^	114.2^bc^	115.9^b^	115.6^b^	0.667	<0.001	<0.001	0.057	0.024
FCR										
1-10 d	1.18	1.18	1.17	1.20	1.15	0.032	0.902	0.918	0.443	0.538
11-24 d	1.44^b^	1.58^ab^	1.54^ab^	1.64^a^	1.51^ab^	0.038	0.011	0.710	0.039	0.288
25-42 d	1.95	2.00	1.96	2.04	1.94	0.040	0.341	0.773	0.113	0.455
1-42 d	1.74^b^	1.81^ab^	1.77^b^	1.86^a^	1.75^b^	0.020	0.001	0.587	0.002	0.130
Mortality, %										
	2.27^c^	10.61^a^	8.33^ab^	11.36^a^	4.55^bc^	1.241	<0.001	0.254	0.002	0.093
EPI	392.0^a^	308.1^cd^	338.6^bc^	303.3^d^	366.0^ab^	7.49	<0.001	0.171	<0.001	0.056

Values are means of 6 pens per treatment combination with 22 male broiler chickens. Means within each row series followed by different lowercase letters differ significantly (*P* < 0.05).

1 NC (negative control), an unchallenged negative control fed a soybean-oil diet and gavaged with sterile saline; PC+SO, a challenged control fed a soybean-oil diet and orally challenged with a mixed *Eimeria* spp. inoculum; PC+SO+CUR, a challenged group fed a soybean-oil diet supplemented with 0.02% curcumin and challenged with a mixed *Eimeria* spp. inoculum; PC+CO, a challenged group fed a canola-oil diet and challenged with a mixed *Eimeria* spp. inoculum; and PC+CO+CUR a challenged group fed a canola-oil diet supplemented with 0.02% curcumin and challenged with a mixed *Eimeria* spp. inoculum.

2 Analyzed as a completely randomized design. Abbreviations: BW, body weight, ADG, average daily gain; ADFI, average daily feed intake; FCR, feed conversion ratio; EPI, European performance index.

In the 2 × 2 factorial analysis (soybean oil vs. canola oil; with vs. without curcumin; excluding the NC), curcumin exerted significant main effects on BW at 24 d (*P* = 0.001) and 42 d (*P* < 0.001), ADG during 11–24 d (*P* = 0.001) and across 1–42 d (*P* < 0.001), and FCR during 11–24 d (*P* = 0.039) and overall 1–42 d (*P* = 0.002). Curcumin also reduced mortality (*P* = 0.002) and increased EPI (*P* < 0.001). In contrast, the main effect of fat source was most evident for ADFI (11–24 d: *P* = 0.002; 25–42 d: *P* = 0.012; 1–42 d: *P* < 0.001), whereas fat source effects on BW, ADG, and FCR were not significant (*P* > 0.05). A fat source × curcumin interaction was detected for ADFI during 11–24 d (*P* = 0.012) and across 1–42 d (*P* = 0.024), indicating that curcumin increased ADFI more clearly in soybean-oil diets than in canola-oil diets; no interactions were observed for BW, ADG, or FCR.

### Hematology

Hematological indices and leukocyte differentials are presented in Table 4. RBC count was lower in the PC+SO group than in the NC (*P* < 0.05), whereas the other challenged groups showed intermediate values and did not differ from the NC. With the exception of PC+CO+CUR, hemoglobin concentration was reduced in all challenged treatments relative to the NC (*P* < 0.05). Hematocrit was also lower in all coccidiosis-challenged groups than in the NC (*P* < 0.05). Total WBC count did not differ among treatments; however, both curcumin-supplemented groups (PC+SO+CUR and PC+CO+CUR) exhibited a lower heterophil proportion and a reduced H/L ratio than the PC+SO group (*P* < 0.05), and were comparable to the NC. Lymphocyte percentage was lower in the PC+SO and PC+CO groups than in the NC (*P* < 0.05).

**Table 4:** Effects of dietary fat source and curcumin supplementation on hematological parameters (on day 24) observed in broiler chickens infected with a mixture of *Eimeria* species at 14 d of age.

	Treatments^1^							*P*-values (2 × 2 factorial design)		
Item	NC	PC+SO	PC+SO+CUR	PC+CO	PC+CO+CUR	SEM	P-values^2^	Fat source	CUR	Fat × CUR
RBC, ×10^6^/µL	2.38^a^	1.84^b^	2.03^ab^	2.03^ab^	2.15^ab^	0.113	0.032	0.195	0.186	0.750
WBC, ×10^3^/µL	21.58	23.14	23.64	24.24	22.86	0.690	0.120	0.796	0.473	0.142
HB, mg/dL	14.67^a^	11.98^b^	12.20^b^	11.92^b^	12.64^ab^	0.495	0.003	0.633	0.318	0.608
Hematocrit, %	31.67^a^	23.50^b^	26.33^b^	23.17^b^	26.67^b^	1.090	<0.001	1.000	0.011	0.772
Leukocyte profile										
Heterophil, %	22.50^b^	29.17^a^	24.00^b^	26.67^ab^	23.83^b^	1.109	0.002	0.220	0.001	0.281
Lymphocyte, %	73.67^a^	64.17^b^	68.83^ab^	66.67^b^	67.83^ab^	1.412	0.001	0.610	0.058	0.241
H/L	0.307^b^	0.460^a^	0.349^b^	0.404^ab^	0.352^b^	0.0234	0.001	0.272	0.003	0.234

Values are means of 6 pens per treatment combination with 2 male broiler chickens. Means within each row series followed by different lowercase letters differ significantly (*P* < 0.05).

1 NC (negative control), an unchallenged negative control fed a soybean-oil diet and gavaged with sterile saline; PC+SO, a challenged control fed a soybean-oil diet and orally challenged with a mixed *Eimeria* spp. inoculum; PC+SO+CUR, a challenged group fed a soybean-oil diet supplemented with 0.02% curcumin and challenged with a mixed *Eimeria* spp. inoculum; PC+CO, a challenged group fed a canola-oil diet and challenged with a mixed *Eimeria* spp. inoculum; and PC+CO+CUR a challenged group fed a canola-oil diet supplemented with 0.02% curcumin and challenged with a mixed *Eimeria* spp. inoculum.

2 Analyzed as a completely randomized design. Abbreviations: RBC, red blood cells, WBC, white blood cells, HB, hemoglobin; H/L, heterophil to lymphocyte ratio.uropean performance index.

In the 2 × 2 factorial analysis, neither the main effect of fat source nor the fat source × curcumin interaction was significant for any hematological variable. In contrast, curcumin supplementation increased hematocrit and decreased heterophil percentage and the H/L ratio (*P* < 0.05).

### Intestinal lesions and oocyst shedding

**Fig. 1:**
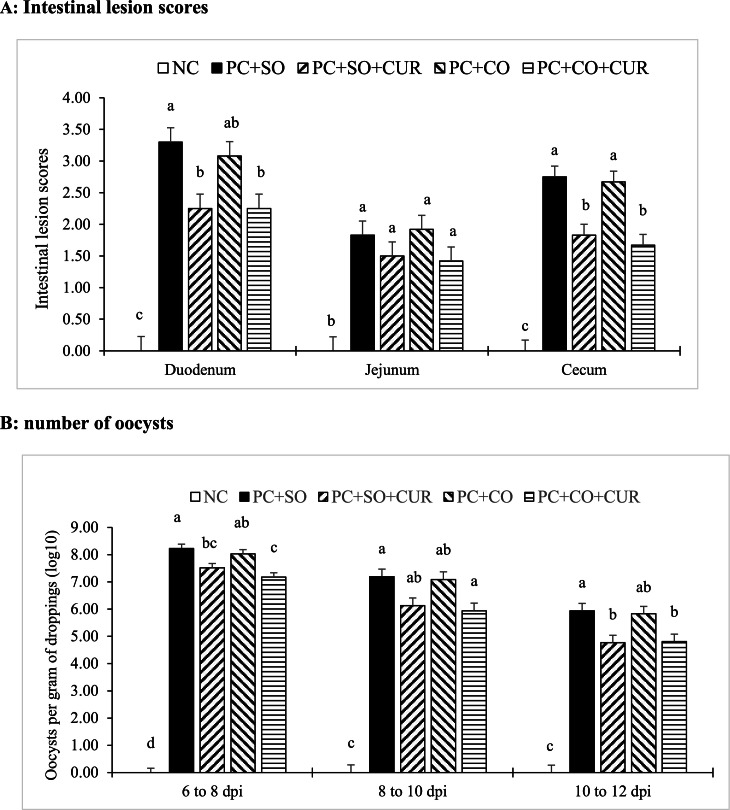
Intestinal lesion scores (A; 6 days after initial infection) and number of oocysts per gram of droppings (B; 6 to 12 days after initial infection) observed in broiler chickens after mixed *Eimeria* species challenge at 14 days of age. Different letters within the same histogram indicate significant differences among groups according to Tukey's multiple-range test (*P* < 0.05). Values are means of 6 replicates (pen) per treatment. The Kruskal-Wallis nonparametric statistical test was used for intestinal lesion scores. The factorial design analysis among challenged groups showed no interaction between fat source and curcumin supplementation in relation to intestinal lesion scores and number of oocysts (*P* > 0.05). Abbreviation: NC (negative control), an unchallenged negative control fed a soybean-oil diet and gavaged with sterile saline; PC+SO, a challenged control fed a soybean-oil diet and orally challenged with a mixed *Eimeria* spp. inoculum; PC+SO+CUR, a challenged group fed a soybean-oil diet supplemented with 0.02% curcumin and challenged with a mixed *Eimeria* spp. inoculum; PC+CO, a challenged group fed a canola-oil diet and challenged with a mixed *Eimeria* spp. inoculum; and PC+CO+CUR a challenged group fed a canola-oil diet supplemented with 0.02% curcumin and challenged with a mixed *Eimeria* spp. inoculum.

Intestinal lesion scores assessed on day 20 (6 days post-infection) are shown in Fig. 1a. No lesions were detected in the NC group. In contrast, the mixed-*Eimeria* challenge induced lesions in the duodenum, jejunum, and cecum (*P* < 0.05). Duodenal lesions associated with *E. acervulina* and cecal lesions associated with *E. tenella* were lower in the curcumin-supplemented groups (PC+SO+CUR and PC+CO+CUR) than in the PC+SO group (*P* < 0.05). Ileal lesions attributed to *E. maxima* did not differ among challenged treatments, although all challenged groups remained higher than the NC (*P* < 0.05).

Fecal oocyst output during days 20–26 (6–12 days post-infection) is summarized in Fig. 1b. No oocysts were detected in the NC group at any sampling point. Relative to PC+SO, both curcumin-supplemented groups (PC+SO+CUR and PC+CO+CUR) exhibited lower oocyst shedding at 6–8 dpi and 10–12 dpi (*P* < 0.05). In addition, PC+CO+CUR showed lower oocyst shedding than PC+CO at 6–8 dpi (*P* < 0.05) and remained lower than PC+SO at 8–10 dpi (*P* < 0.05), whereas the remaining challenged treatments were generally intermediate.

In the 2 × 2 factorial analysis, neither the main effect of fat source nor the fat source × curcumin interaction influenced lesion scores or oocyst shedding (*P* > 0.05). In contrast, curcumin exerted significant main effects by reducing lesion scores in the duodenum and cecum and decreasing oocyst shedding across post-challenge intervals (*P* < 0.05), whereas its effect on ileal lesion scores was not significant.

### Nutrient digestibility

Nutrient digestibility coefficients and AMEn at 24 d are presented in Table 5. Dry matter digestibility and AMEn were higher in the NC group than in the PC+SO group (*P* < 0.05), whereas the remaining challenged treatments exhibited intermediate values and did not differ from the NC. Ash digestibility tended to be lower in the PC+SO and PC+CO groups than in the NC (*P* = 0.079). The AID of crude protein and crude fat was not affected by treatment (*P* > 0.05).

**Table 5:** Effects of dietary fat source and curcumin supplementation on nutrient digestibility (on day 24) observed in broiler chickens infected with a mixture of *Eimeria* species at 14 d of age.

	Treatments^1^							*P*-values (2 × 2 factorial design)		
Item	NC	PC+SO	PC+SO+CUR	PC+CO	PC+CO+CUR	SEM	*P*-values^2^	Fat source	CUR	Fat × CUR
DM, %	77.74^a^	74.95^b^	76.03^ab^	75.59^ab^	76.16^ab^	0.550	0.021	0.509	0.163	0.653
CP, %	72.05	69.88	71.58	70.07	71.65	0.773	0.186	0.848	0.034	0.919
CF, %	80.77	78.69	78.82	79.84	80.39	0.715	0.191	0.087	0.630	0.776
Ash, %	51.10	48.84	50.47	48.25	50.65	0.798	0.079	0.820	0.027	0.643
AMEn, kcal/kg	3070^a^	3022^b^	3039^ab^	3040^ab^	3049^ab^	8.847	0.014	0.140	0.196	0.679

Values are means of 6 pens per treatment combination with 2 male broiler chickens. Means within each row series followed by different lowercase letters differ significantly (*P* < 0.05).

1 NC (negative control), an unchallenged negative control fed a soybean-oil diet and gavaged with sterile saline; PC+SO, a challenged control fed a soybean-oil diet and orally challenged with a mixed *Eimeria* spp. inoculum; PC+SO+CUR, a challenged group fed a soybean-oil diet supplemented with 0.02% curcumin and challenged with a mixed *Eimeria* spp. inoculum; PC+CO, a challenged group fed a canola-oil diet and challenged with a mixed *Eimeria* spp. inoculum; and PC+CO+CUR a challenged group fed a canola-oil diet supplemented with 0.02% curcumin and challenged with a mixed *Eimeria* spp. inoculum.

2 Analyzed as a completely randomized design. Abbreviations: DM, dry matter, CP. Crude protein; CF, crude fat; AMEn, apparent metabolizable energy corrected for nitrogen retention.

In the 2 × 2 factorial analysis, no fat source × curcumin interaction was detected for any digestibility variable or AMEn (*P* > 0.05). A tendency for greater fat digestibility was observed in canola-oil diets compared with soybean-oil diets (*P* = 0.087). Curcumin supplementation increased crude protein digestibility and ash digestibility (*P* < 0.05).

### Gut morphology

Intestinal morphometric indices at 24 d are presented in Table 6. In the duodenum, the PC+SO and PC+CO treatments exhibited reduced VH and VSA, together with increased CD, compared with the NC (*P* < 0.05). All challenged groups also showed a lower VH/CD ratio than the NC (*P* < 0.05). Duodenal GC counts in PC+CO+CUR were comparable to the NC and higher than in PC+SO, whereas the NC also exceeded PC+SO+CUR and PC+CO (*P* < 0.05). In the jejunum, VH was lower in PC+SO than in the NC (*P* < 0.05). In addition, PC+SO and PC+CO had lower VH/CD ratio, VSA, and GC counts than the NC (*P* < 0.05), whereas the remaining treatments were intermediate and did not differ from the NC. In the ileum, VH and VSA were reduced in PC+SO and PC+CO relative to the NC (*P* < 0.05). GC counts were highest in the NC among the challenged treatments, and PC+CO+CUR increased ileal GC counts compared with PC+SO (*P* < 0.05).

**Table 6:** Effects of dietary fat source and curcumin supplementation on serum antioxidant status and corticosterone concentration (on day 24) observed in broiler chickens infected with a mixture of *Eimeria* species at 14 d of age.

	Treatments^1^							*P*-values (2 × 2 factorial design)		
Item	NC	PC+SO	PC+SO+CUR	PC+CO	PC+CO+CUR	SEM	*P*-values^2^	Fat source	CUR	Fat × CUR
Duodenum										
VH, µm	1561^a^	1218^b^	1334^ab^	1257^b^	1358^ab^	59.3	0.004	0.621	0.098	0.909
VW, µm	174.9	166.2	171.8	163.6	169.1	7.95	0.866	0.738	0.503	0.994
CD, µm	192.8^b^	295.9^a^	273.9^ab^	290.6^a^	251.2^ab^	21.32	0.014	0.428	0.092	0.620
VH/CD	8.89^a^	4.29^b^	4.89^b^	4.37^b^	5.48^b^	0.534	<0.001	0.336	0.021	0.459
VSA^3^, mm	0.853^a^	0.635^b^	0.720^ab^	0.640^b^	0.721^ab^	0.0398	0.004	0.942	0.066	0.954
GC, n/villus	114.5^a^	72.6^c^	93.0^bc^	75.9^bc^	95.5^ab^	5.15	<0.001	0.559	0.001	0.929
Jejunum										
VH, µm	1369^a^	1152^b^	1268^ab^	1175^ab^	1261^ab^	48.1	0.029	0.849	0.022	0.720
VW, µm	179.2	158.2	169.2	159.7	165.9	9.64	0.557	0.893	0.193	0.715
CD, µm	272.8	293.1	273.2	286.0	271.5	7.43	0.180	0.567	0.033	0.727
VH/CD	5.04^a^	3.94^b^	4.65^ab^	4.12^b^	4.66^ab^	0.199	0.005	0.578	0.002	0.624
VSA^3^, mm	0.755^a^	0.571^b^	0.673^ab^	0.590^b^	0.657^ab^	0.0367	0.012	0.959	0.015	0.598
GC, n/villus	127.1^a^	85.1^b^	104.7^ab^	91.7^b^	107.7^ab^	5.80	0.000	0.403	0.005	0.743
Ileum										
VH, µm	1179^a^	956^b^	1079^ab^	974^b^	1096^ab^	37.7	0.002	0.656	0.004	0.987
VW, µm	178.8	173.8	177.0	171.0	176.5	6.53	0.924	0.811	0.532	0.867
CD, µm	260.4	273.2	271.4	268.9	269.2	8.32	0.841	0.691	0.925	0.898
VH/CD	6.63	5.54	6.16	5.78	6.22	0.323	0.185	0.653	0.125	0.809
VSA^3^, mm	0.662^a^	0.521^b^	0.597^ab^	0.523^b^	0.609^ab^	0.0276	0.006	0.813	0.009	0.868
GC, n/villus	96.5^a^	68.9^c^	78.1^bc^	70.7^bc^	81.4^b^	2.83	<0.001	0.359	0.001	0.781

Values are means of 6 pens per treatment combination with 2 male broiler chickens. Means within each row series followed by different lowercase letters differ significantly (*P* < 0.05).

1 NC (negative control), an unchallenged negative control fed a soybean-oil diet and gavaged with sterile saline; PC+SO, a challenged control fed a soybean-oil diet and orally challenged with a mixed *Eimeria* spp. inoculum; PC+SO+CUR, a challenged group fed a soybean-oil diet supplemented with 0.02% curcumin and challenged with a mixed *Eimeria* spp. inoculum; PC+CO, a challenged group fed a canola-oil diet and challenged with a mixed *Eimeria* spp. inoculum; and PC+CO+CUR a challenged group fed a canola-oil diet supplemented with 0.02% curcumin and challenged with a mixed *Eimeria* spp. inoculum.

2 Analyzed as a completely randomized design. Abbreviations: VH, villus height; VW, villus width; CD, crypt depth; VH/CD, villus height to crypt depth ratio; VSA, villus surface area; GC, goblet cell.

In the 2 × 2 factorial analysis, neither fat source nor the fat source × curcumin interaction significantly affected small-intestinal morphometric traits (*P* > 0.05). In contrast, curcumin supplementation increased VH (jejunum and ileum), VH/CD ratio (duodenum and jejunum), VSA (jejunum and ileum), and GC counts (duodenum, jejunum, and ileum), while reducing CD in the jejunum (*P* < 0.05).

### Tight-junction gene expression

**Fig. 2:**
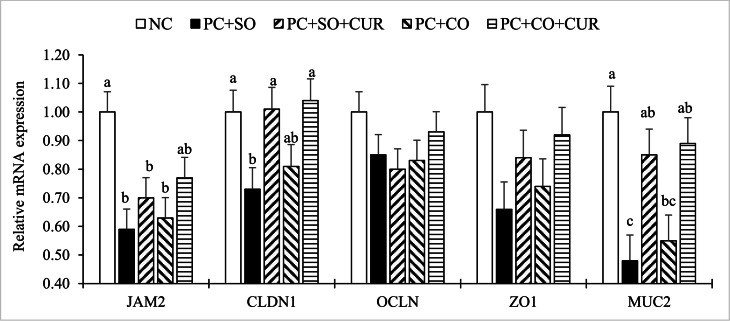
Bar charts of jejunal mRNA expression levels of junctional adhesion molecule 2 (**JAM2**), claudin 1 (**CLDN1**), occludin (**OCLN**), zonula occludens 1 (**ZO1**), and mucin 2 (**MUC2**) in 24-days-old broiler chickens following a mixed *Eimeria* species challenge at 14 days of age. Different letters within the same histogram indicate significant differences among groups according to Tukey's multiple-range test (*P* < 0.05). Values are means of 6 replicates (pen) per treatment and 2 chickens per replicate. The factorial design analysis among challenged groups showed no interaction between BAG and MPH in relation to jejunal mRNA expression levels of tight-junction proteins (*P* > 0.05). Abbreviation: NC (negative control), an unchallenged negative control fed a soybean-oil diet and gavaged with sterile saline; PC+SO, a challenged control fed a soybean-oil diet and orally challenged with a mixed *Eimeria* spp. inoculum; PC+SO+CUR, a challenged group fed a soybean-oil diet supplemented with 0.02% curcumin and challenged with a mixed *Eimeria* spp. inoculum; PC+CO, a challenged group fed a canola-oil diet and challenged with a mixed *Eimeria* spp. inoculum; and PC+CO+CUR a challenged group fed a canola-oil diet supplemented with 0.02% curcumin and challenged with a mixed *Eimeria* spp. inoculum.

Jejunal expression of tight-junction– and barrier-related markers is shown in Fig. 2. Expression of OCLN and ZO1 did not differ among treatments (*P* > 0.05). In contrast, JAM2 expression was lower in the challenged groups fed PC+SO, PC+SO+CUR, or PC+CO than in the NC (*P* < 0.05), whereas PC+CO+CUR exhibited an intermediate response. For the barrier- and mucus-associated genes, CLDN1 and MUC2 expression was markedly reduced in the PC+SO group; however, curcumin supplementation (PC+SO+CUR and PC+CO+CUR) increased CLDN1 and MUC2 to levels comparable with the NC and higher than PC+SO (*P* < 0.05).

In the 2 × 2 factorial analysis, neither the main effect of fat source nor the fat source × curcumin interaction was significant for any measured transcript (*P* > 0.05). By contrast, curcumin exerted significant main effects on CLDN1 and MUC2, indicating that supplementation upregulated these genes irrespective of oil source (*P* < 0.05).

## Discussion

Across the grow-out period, the coccidiosis challenge caused a coordinated decline in performance, with lower BW and ADG, poorer feed efficiency, and higher mortality. This pattern is consistent with evidence that *Eimeria* disrupts epithelial integrity, limits nutrient capture, and increases the nutrient and energy costs of immune activation (Ahmad et al., 2023; Mathis et al., 2025). The absence of differences during 1–10 d (Table 3) suggests that early growth was still largely driven by hatch-related physiology and the high digestibility of starter diets, whereas treatment separation emerged once intestinal injury and inflammatory pressure became more influential. Notably, the PC+CO+CUR group showed the best overall performance among challenged treatments, with higher BW and ADG, lower mortality, and the highest EPI, indicating a meaningful flock-level advantage. The overall pattern of performance responses supports an efficiency-based interpretation. Soybean oil–based diets generally exhibited lower ADFI and lower ADG, whereas PC+CO+CUR improved growth and overall efficiency without a corresponding increase in ADFI, a pattern more consistent with enhanced nutrient utilization than with simple appetite stimulation. Factorial analysis also detected a significant fat source × curcumin interaction for ADFI during 11–24 d and across 1–42 d, implying that curcumin’s effect on intake depended on the lipid matrix; however, because this interaction was not significant for BW, ADG, or FCR, curcumin’s growth and efficiency benefits appeared broadly consistent across fat sources, and the interaction mainly refined interpretation of intake regulation. Collectively, these results align with evidence that phytogenic feed additives help sustain productive performance during enteric stress by stabilizing digestive and absorptive function and reducing inflammatory inefficiency (Abdelli et al., 2021; Wang et al., 2024). The findings are also consistent with curcumin attenuating key pathological features of coccidiosis that undermine performance, including epithelial injury, barrier disruption, oxidative stress, and inflammatory dysregulation (Li et al., 2024; Mathis et al., 2025; Oke et al., 2024). In *Eimeria*-challenged broilers, curcumin has similarly been associated with improved growth, stronger antioxidant defenses, and better intestinal integrity, potentially through modulation of gut microbiota, microbial metabolites, and inflammation-related gene expression (Chen et al., 2024a,b). Complementary evidence indicates that curcumin can enhance growth and anti-inflammatory function through modulation of the gut microbiota, microbiota-derived metabolites, and inflammation-related gene expression, providing a coherent link between gut ecology and improved efficiency (Chen et al., 2024a). The observed dependence of ADFI on fat source is biologically plausible because dietary lipids can alter microbial composition and predicted function, thereby influencing fermentation products and the barrier environment (Jadhav et al., 2024; Li et al., 2024). Finally, given that PC+CO+CUR was the strongest-performing treatment among the challenged groups, it is plausible that this relative advantage could partly reflect fat-matrix–related differences in curcumin bioavailability (i.e., delivery and absorption kinetics), a possibility increasingly discussed in work using formulation approaches such as emulsion-based protection (Bertoncini-Silva et al., 2024; Khosinklang et al., 2026).

The erythrogram at day 24 indicates that the coccidiosis challenge reduced blood oxygen-carrying capacity, reflected by lower hemoglobin and hematocrit across challenged treatments and the most evident RBC depression in the PC+SO group (Table 4). This pattern is consistent with the systemic burden of coccidiosis, in which intestinal injury and inflammation can impair nutrient assimilation and intensify inflammatory/oxidative pressure, producing anemia-like shifts in hematological indices (Ahmad et al., 2023; Mathis et al., 2025). Similar changes have been reported in challenged broilers, supporting the use of erythrocyte- and hemoglobin-related measures as indicators of enteric disruption and overall physiological cost (Ghasemi et al., 2025). The comparatively better hemoglobin status in PC+CO+CUR aligns with evidence that curcumin can attenuate infection-associated oxidative and inflammatory disturbances during *Eimeria* challenge, and that improved curcumin delivery can amplify systemic responses (Chen et al., 2024b; Fathi et al., 2025). Total WBC count was unchanged, but leukocyte differentials were more informative: non-curcumin challenged groups showed higher heterophils and H/L ratio with lower lymphocytes, consistent with H/L as an integrative stress and immune-redistribution marker in poultry (Ghasemi and Nari, 2023; Hashim et al., 2023) and as a trait linked to immune robustness and disease responsiveness (Zhang et al., 2023). Curcumin shifted profiles toward the unchallenged NC by reducing heterophils and H/L. Factorial analysis reinforced this conclusion, with no fat-source or interaction effects but significant main effects of curcumin (higher hematocrit; lower heterophils and H/L).

The lesion and oocyst data collectively indicate that the dietary strategy influenced both mucosal injury and parasite replication, two tightly coupled outcomes in coccidiosis. Lesion scores capture local epithelial destruction during intracellular development, whereas oocyst shedding is a practical indicator for reproductive success and transmission pressure; concomitant reductions in both therefore support true biological mitigation rather than a purely symptomatic effect (Bafundo, 2025; Santos et al., 2024). Importantly, oocyst counts collected during 6–8 dpi likely represent primary shedding more closely, whereas counts obtained during 8–10 and 10–12 dpi under floor-pen conditions may also reflect secondary cycling and reinfection. Therefore, the later intervals should be interpreted as indicators of overall pen-level shedding dynamics rather than strictly first-cycle parasite output. In this context, curcumin supplementation consistently reduced duodenal and cecal lesion severity and was accompanied by lower oocyst output across multiple post-infection intervals, suggesting that curcumin limited parasite amplification and the resulting tissue damage (Chen et al., 2024b; Lee et al., 2025). Similar profiles, reduced lesions together with lower oocyst excretion and improved gut integrity signals, have been reported for phytogenic and multi-botanical interventions under *Eimeria* challenge, reinforcing that suppressing early replication while moderating downstream inflammation can measurably lessen intestinal pathology and environmental contamination (Galamatis et al., 2025; Han et al., 2022; Nooreh et al., 2021).

Mechanistically, the curcumin-associated reductions in lesion scores and shedding are consistent with a multi-target mode of action integrating antioxidant support, inflammatory modulation, and barrier preservation, which collectively may reduce the efficiency with which *Eimeria* colonizes and damages the epithelium (Fouad et al., 2025; Sureshbabu et al., 2023). Curcumin has been linked to stronger antioxidant defenses and attenuated pro-inflammatory signaling in challenged birds, effects that could limit epithelial loss and crypt dysfunction that otherwise facilitate parasite cycling and prolong shedding (Petrone-Garcia et al., 2021). The site-specific nature of the response is also notable: curcumin improved lesions in the duodenum and cecum but not in the jejunum, which may reflect species- and intestinal segment–specific differences in parasite biology and lesion dynamics, including the greater variability typically associated with *E. maxima* pathology. Finally, although curcumin is lipophilic and could plausibly exhibit fat-dependent bioavailability, factorial analysis detected neither a fat-source main effect nor a fat source × curcumin interaction for lesions or oocyst shedding, indicating that curcumin’s anticoccidial effects were largely robust across the two oil matrices under the present dietary conditions.

The digestibility results indicate that the enteric challenge reduced nutrient extraction efficiency, with the clearest separation observed between the NC and PC+SO groups for DM digestibility and AMEn. This pattern is consistent with coccidiosis pathophysiology, in which epithelial damage, inflammatory exudation, and increased endogenous losses depress apparent digestibility and available energy (Alagbe et al., 2024; Gulizia et al., 2025). The intermediate responses in the curcumin-supplemented treatments suggest partial functional recovery rather than full normalization by day 24, which is biologically plausible because these measurements were obtained 10 days after challenge, when birds were likely transitioning from the peak acute phase toward early recovery rather than peak lesion expression. Accordingly, the digestibility and related physiological responses reported here should be interpreted as reflecting the integrated consequences of earlier epithelial injury together with ongoing mucosal repair and metabolic reprioritization under floor-pen conditions, where limited recycling of oocysts may also occur. It is therefore plausible that the magnitude of some responses would have differed if sampling had been conducted earlier, particularly during the 5–7 days post-challenge window, when primary intestinal damage is generally expected to be greatest (Alagbe et al., 2024). Factorial analysis refined these inferences by showing significant main effects of curcumin on CP and ash digestibility, with no Fat × CUR interaction, indicating that curcumin’s benefits on protein and mineral utilization were expressed across both oil sources. This response aligns with evidence that phytogenic compounds can improve digestive efficiency under intestinal stress by supporting mucosal integrity, moderating oxidative and inflammatory pressure, and favorably influencing digestive physiology (Alharbi et al., 2025; Obianwuna et al., 2024). Consistent with this, a recent meta-analysis linked dietary curcumin to improved antioxidant status and intestinal morphology, providing a plausible bridge between reduced gut inefficiency and higher apparent utilization of protein and minerals (Hernández-García et al., 2025). The near-significant tendency for higher fat digestibility in canola-oil diets is also mechanistically coherent, as lipid digestion relies on emulsification, micelle formation, and bile–lipase dynamics that vary with fatty-acid profile and can become more limiting during enteric insults (Oketch et al., 2023).

Coccidiosis disrupts intestinal mucosal structure by damaging the epithelium, increasing cell turnover, and shifting resources from growth to repair and immune responses (Chen et al., 2025; Su et al., 2024). Accordingly, the challenged PC+SO and PC+CO groups showed intestinal damage across segments, including shorter villi, reduced villus surface area, deeper crypts, and a lower VH/CD ratio, especially in the duodenum and ileum. These changes indicate reduced absorptive capacity and greater energetic demands for epithelial renewal (de Souza et al., 2024; Su et al., 2024). Lower GC counts in challenged birds further suggest impaired mucus protection, which may weaken barrier function and favor secondary dysbiosis (Chen et al., 2025; Goo et al., 2023). Consistently, MUC2 expression was most reduced in the PC+SO group and was restored toward NC levels by curcumin (Fig. 2), supporting the conclusion that challenge compromised the mucus layer (Al-Baadani et al., 2025; Goo et al., 2023).

Curcumin supplementation partially offset these structural costs, with curcumin-containing diets shifting villus architecture and mucus-associated defenses toward the unchallenged phenotype, particularly by improving VH/CD ratio and GC numbers, and increasing VH and VSA in the jejunum and ileum. These responses are consistent with evidence that curcumin supports epithelial integrity by limiting oxidative injury and excessive inflammatory activation, thereby reducing crypt hyperplasia and helping preserve villus structure (Chen et al., 2024b; Hernández-García et al., 2025). Notably, the factorial analysis detected no fat-source main effect and no fat source × curcumin interaction, indicating that the morphologic benefits of curcumin were expressed similarly across oil sources and were not contingent on a specific lipid matrix. This strengthens the inference that curcumin’s primary contribution was mucosal protection under infection stress. Recent poultry literature likewise reports that curcumin can improve villus indices and barrier-related outcomes under stressors, providing a plausible pathway linking improved mucosal structure with better nutrient capture and disease resilience (Fouad et al., 2025; Khosinklang et al., 2026).

The transcriptional profile (Fig. 2) suggests that, at the mucosal level, coccidiosis primarily impaired epithelial sealing and mucus-barrier signaling rather than uniformly suppressing all tight-junction transcripts. The downregulation of CLDN1 and MUC2 in the soybean-oil challenged group is especially informative because claudins regulate paracellular selectivity and MUC2 supports the mucus layer that separates luminal contents from the epithelium (Hossain et al., 2026; Kuo et al., 2022; Tan et al., 2025). The lack of treatment effects on OCLN and ZO1 does not preclude barrier disruption, since tight-junction injury may also reflect protein redistribution, phosphorylation, and cytoskeletal remodeling, whereas the reduction in JAM2 across most challenged diets further supports junctional disturbance (Criado-Mesas et al., 2021; Hossain et al., 2026; Kuo et al., 2022). Curcumin shifted this response toward a more protected mucosal phenotype, as CLDN1 and MUC2 were restored in both curcumin-supplemented groups, consistent with reports that turmeric/curcumin supports epithelial defense under intestinal stress (Fouad et al., 2025; Xu et al., 2024; Zhang et al., 2025). Notably, factorial analysis detected neither a fat-source main effect nor a fat source × curcumin interaction, indicating that curcumin-associated upregulation of CLDN1 and MUC2 was expressed similarly in soybean- and canola-oil diets. Biologically, this consistency suggests that curcumin’s barrier-supporting signal at the transcript level is relatively robust across practical oil sources, even though the challenge continued to depress other junction-related markers such as JAM2. One plausible interpretation is that curcumin preferentially reinforces first-line protective elements, mucin production and key sealing components, thereby limiting antigen leakage and dampening downstream inflammatory demand (Kuo et al., 2022; Tan et al., 2025), a pathway emphasized in current models of tight-junction remodeling during intestinal inflammation.

## Conclusion

A mixed-*Eimeria* challenge caused a clear gut-to-performance penalty in broilers, increasing lesion scores and oocyst shedding, compromising intestinal morphology and barrier function (reduced villus development and goblet cells, lower CLDN1/MUC2), worsening hematological stress indicators, reducing nutrient utilization, and impairing growth efficiency, livability, and EPI. Curcumin was the dominant effective factor, reducing lesions and shedding, supporting mucosal recovery and barrier-related signaling, improving protein and mineral utilization, and ultimately improving flock performance under challenge. Fat source had minor effects and did not meaningfully modify curcumin responses. Overall, 0.02% curcumin is a practical nutrition-based strategy to improve resilience to coccidiosis by limiting parasite-associated mucosal injury and promoting barrier recovery. Future studies should optimize dose and delivery and confirm mechanisms using permeability assays, and lesion kinetics across multiple post-infection time points.

## CRediT authorship contribution statement

**Hussein Maytham Abdulhusein:** Writing – review & editing, Writing – original draft, Investigation, Data curation, Conceptualization. **Kamran Taherpour:** Writing – review & editing, Writing – original draft, Supervision, Software, Project administration, Formal analysis, Conceptualization. **Hossein Ali Ghasemi:** Writing – review & editing, Writing – original draft, Supervision, Methodology, Formal analysis. **Hassan Shirzadi:** Writing – review & editing, Validation, Resources, Methodology. **Fatemeh Tavakolinasab:** Writing – review & editing, Visualization, Software, Investigation.

## Disclosures

All authors approve the submission of this manuscript and declare no conflict of interest.

## Appendix Supplementary materials

Image, application 1

## Footnotes

Supplementary material associated with this article can be found, in the online version, at doi:10.1016/j.psj.2026.106981.
