# Supplementary material for: Curcumin-mediated resilience to mixed Eimeria challenge in broilers fed soybean or canola oil: growth performance, hematology, coccidial lesions, oocyst shedding, digestibility, and intestinal barrier biology
Source: Poult Sci. 2026 Apr 19;105(8):106981. doi: 10.1016/j.psj.2026.106981 (PMC13156625; doi:10.1016/j.psj.2026.106981)
Supplement: Supplementary file 1 [file mmc1.pdf]

Table S1. Analyzed gross energy, fat content, and fatty acid profile of basal starter, grower, and finisher diets fed to broiler chickens in different growing phases

	Starter[1]		Grower		Finisher	
Ingredient (%)	SO	CO	SO	CO	SO	CO
Gross energy (kcal/kg)	4320	4328	4482	4490	4576	4582
Crude fat (%)	6.32	6.40	6.55	6.67	6.78	6.87
Fatty acid profile (% of total fatty acids)						
C16:0	11.34	7.25	11.33	7.40	11.34	7.44
C18:0	3.47	1.82	3.40	1.79	3.33	1.76
C18:1n-9	25.55	44.22	25.67	43.88	25.79	43.53
C18:2n-6	55.58	39.77	55.60	40.18	55.62	40.60
C18:3n-3	2.75	4.58	2.67	4.45	2.58	4.32
n-6/n-3 PUFA	20.21	8.68	20.82	9.03	21.55	9.40

[1] SO, soybean oil; CO, canola oil.

Table S2. Gene special primers (tight junction genes and MUC2) for quantitative real-time PCR.

Gene[1]	Primer sequence (5⁰–3⁰)[2]	Length (nt)	GenBank number
OCLN	F: GGCCACCATGTTCAGCAAGAA<br>R: GACCCGTAGCCGTAATCAGCC	99	XM_046904540.1
CLDN1	F: AGAGGCATCAGGTATCTGGGT<br>R: CCCATCGAGAAGTAGGAGCCA	214	NM_001013611.2
JAM2	F: GGATTCTGGGACCTACCGCTG<br>R: CTGCCTGTTCCTGTCTTTTCC	243	XM_046907882.1
ZO1	F: CCCTGCCCGTGGGATGTTT<br>R: GCCCTGGCAGACATTTTGTTT	138	XM_040680630.2
MUC2	F: CTGATTGTCACTCACGCCTTAATC<br>R: GCCGGCCACCTGCAT	147	JX284122.1
GAPDH	F: CAGAACATCATCCCAGCGTCCAC<br>R: CGGCAGGTCAGGTCAACAACAG	134	NM_204305.2

[1] OCLN = occludin; CLDN1 = claudin1; JAM2 = junctional adhesion molecule 2; ZO1 = zonula occluden 1; MUC2, mucin 2; GAPDH = glyceraldehyde-3-phosphate.

[2] F = forward primer; R = reverse primer.
